# Security of passenger transport in the Baltic Sea in the context of foreign terrorist fighters

**DOI:** 10.1007/s12198-020-00213-3

**Published:** 2020-07-08

**Authors:** Katarzyna Wardin

**Affiliations:** Polish Naval Academy of the Heroes of Westerplatte, Śmidowicza Street, 69 81-127 Gdynia, Poland

**Keywords:** Security, Passenger transportation, Terrorism, Foreign fighters

## Abstract

The Baltic Sea basin is one of the busiest areas in Europe in terms of the passenger transport and is considered as a domestic sea of the European Union, very important for the development and prosperity of all citizens. A high number of ferries connections along with exclusive cruise ships, visiting the Baltic countries, make the sea very busy. At the same time the security of lines of communications has become the highest priority, especially that a possibility of terrorist attacks still poses a serious threat. Although terrorism has been being fought intensively since 11th September, terrorist organizations such as Al-Qaeda or Islamic State of Iraq and the Levant, are still able to attack. The article considers the threat of organizing a terrorist attack posed by Foreign Terrorist Fighters returning home to the Baltic countries. Due to the fairly high number of Foreign Terrorist Fighters in some countries around the Baltic Sea, very intense passenger traffic in the area, and the appeals which have been proclaimed by Al-Qaeda and Islamic State leaders, there is a possibility of a terrorist attack occurrence in the near future. Cited facts lead to the conclusion that some precautions should be taken both in terms of technical and organizational measures to make passenger traffic secure.

## Introduction

The activities of terrorist organizations around the world have been a threat to security since antiquity. In the second half of the twentieth century, it assumed an international dimension, and since the beginning of the twenty-first century, terrorism has been classified as a global threat, mainly due to the tragic terrorist attacks of September 11, 2001 on the World Trade Center and the Pentagon in the United States, organized by the terrorist organization Al-Qaeda. This is not the only international terrorist organization that in the twenty-first century has the capabilities and has set itself the goal of attacking Western countries. Islamic State of Iraq and the Levant (ISIL) is an organization operating until recently in part of the territory of Syria and Iraq, which as a result of the civil war in Syria since the beginning of the so-called The Arab Spring has caused an influx of fighters - jihadists - from various countries around the world including Western countries, as well as European ones. Due to the activities of the international counterterrorist coalition in 2018, ISIL transferred its cell operations to Libya, destabilized by civil war. The most worrying is the fact that some of the fighters who came to Syria to fight for ISIL with European passports returned to their countries as so-called Foreign Terrorists Fighters (FTF) and they can pose a serious threat to the security of Europeans. The return of them has taken place recently which explains very little studies in terms of reports, scientific papers and none of the books. If they are available they concern only general threat of terrorists in the Baltic Sea area.

Maritime terrorism has not been the domain of terrorist activity so far, although such attacks have occurred in the past. Examples are the attacks on *MS Achille Lauro* in 1985 in the Mediterranean Sea, the bomb attack on *SuperFerry 14* in the Philippines in 2004. Organizing an act of maritime terrorism requires specific preparations, but this does not mean that in the near future terrorists will not focus on attacking the “soft underbelly” of the richest countries in the world, i.e. sea transport, with particular emphasis on passenger transport, including ro-ro sea ferries and exclusive passenger ships. The Baltic Sea basin, due to its climate, is the most attractive for external tourist passenger traffic in the summer months from April to September, but for countries located on the Baltic Sea, transport in general, including ferry ro-ro is an important component of the economic success of these countries and 85 million Europeans living on the Baltic Sea or related to the economy. As long as people have lived in the area, the Baltic Sea has provided a strong connection between the countries and a source of human livelihood (HELCOM 2017). The Baltic is known for its ferry and ro-ro shipping intensity and the majority of the intra-Baltic Sea Region (BSR) trade passes through Baltic ports. Ro-ro and ferry traffic goes through 71 ports within the borders of the BSR, with more than half of them in Sweden, Denmark and Finland. The Baltic is linked directly with at least 30 ports located in the rest of Europe (Kłopott 2017). Apart from that there is also still a growing number of passengers visiting the Baltic Sea ports during shorter visits of cruise liners. The above information is the basis for drawing the conclusion that the intensive transport of ro-ro, ro-pax passenger ferries in the Baltic Sea basin is an important economic element and therefore the author speculates that due to its intensity and FTF return to European countries, it may become the target of terrorist activities.

In terms of methodology the estimation of the rising threat of terrorist attacks presented in the article is based on a four-step approach. First, a current situation of Al-Qaeda and ISIL is described. Secondly, the number of returning TFT is analyzed based on available data and information. Thirdly, the ro-pax, ro-ro passenger ferries and cruise lines in the BSR are presented. Finally the author estimates a rising threat of a terrorist attack on passenger transport through analysis and synthesis of facts and statistical data. All that allows to conclude that to keep BSR safe and secure ports’ authorities should take same precautions. The author suggests two ways actions.

The problem discussed in the article is not new but it does not have many references in the literature. The BSR is well described in terms of safety and security in general as well as analyses of passenger transport, both ferries and cruise. The most valid reports and articles used in the article *Beyond the Caliphate: Foreign Fighters and the Threat of Returnees*, by Richard Barrett which helped to evaluate the potential threat of TFTs and *Baltic LINes: Shipping in the Baltic Sea – Past, present and future developments relevant for Maritime Spatial Planning*, by Nele Kristin Meyer and Megalist of Baltic Sea Ferry Operators 2019, *Cruise ports of call in the Baltic.* These helped to estimate the size of the passenger transport. Because there have not been any terrorist attacks on maritime traffic in the BSR so far, the literature in this subject is not present. That was the main reason why the author decided to look closer into this matter and analyze the potential threat of such activities and to give some possible solutions.

## The condition of al-QAEDA and ISIL at the end of 2018

The most dangerous terrorist organization that has committed itself in the past and still has the ability to organize terrorist attacks in Western countries is the international network called Al-Qaeda. It was created in 1988 by Abd Allah Azzam and its co-creator and later leader was Osama bin Laden. Al-Qaida is responsible for attacks on the World Trade Center and the Pentagon in the USA as well as for attacks that took place after September 11 in European countries. Since those tragic events, the activities of terrorist organizations have been one of the most serious global threats of the twenty-first century. Over the past 45 years since 1970, there have been over 16,000 terrorist acts in Western Europe. They were carried out by both organized groups and individual terrorists of various ideologies. However, over the past 20 years, attacks organized by Islamic terrorists have gained special significance. Over 630 Europeans were killed in the terrorist attacks from 2001 to mid-2017 (Stanley 2016, Global Terrorism Index 2016).

Al-Qaeda still remains a very dangerous organization that has found space to rebuild its influence in Libya destabilized by the civil war. Two branches: the Islamic Maghreb Al-Qaida and the Arabian Peninsula Al-Qaida, play a special role here, both groups support financially and provide logistical, tactical and training support to the Libyan terrorist group Ansar al Sharia, treating Libya as its own base. This manifests itself in the use of Libyan territory to plan an attack on strategic goals, including the preparation of attacks on other theatres of activity, especially against regimes in northern Africa. Al-Qaida Islamic Maghreb uses the southwestern region of Libya as a base to coordinate regional operations.

Grand Strategic Objectives are still consistent:

• Unify the global Salafi-jihadi movement under al Qaeda’s leadership.

• Expel the West and Russia from Muslim lands.

• Transform Muslim societies to establish Islamic polities that reflect al Qaeda’s ideology (Estelle 2017).

Support provided by the organization to the Ansar al Sharia group and other smaller and local councils, the shuras, enables high-level terrorist activities.

Al-Qaeda leaders are aware of the fact that ISIL fighters are excellent and fresh material/new blood for organizations that have ideologically been extremely successful and can be used for the needs of the organization’s activities both in Libya itself and in Europe. Although Libya after the civil war can in some sense be compared to “no man’s land”, Al-Qaeda did not gain a certain position there, and it is rather dependent on the course of cooperation with local people and smaller organizations. For now, the prevailing chaos in Libya has a positive effect on strengthening Al-Qaeda’s position.

The rise and development of ISIL is of particular concern in the context of terrorist attacks on the European continent. Its beginning dates back to 1999 when Abu Musab al-Zarqawi founded the Organization of Monotheism and Jihad - Jama’at al-Tawhid wal-Jihad, which was an armed jihadist group (Stachota 2015). It operated in Jordan, Afghanistan and Iraq, where it was in fact a branch of Al-Qaeda. The leader declared war in 2004 not only on the states intervening in Iraq, but also on the Shiites who gained power in the country thanks to the intervention of the United States after the overthrow of the Sunni regime of Saddam Husain, thus driving Iraq into a vortex of religious war. In the same year, it passed under the informal control of Osama bin Laden, and the cooperation of both organizations resulted in new recruits and the strengthening of the financial and logistics base of activities (Hashim 2009).

In 2006, after the death of Abu Musab al-Zarqawi, the emergence of the Islamic State of Iraq was announced, but the consolidated activities of the coalition of Sunni tribes, former military, supported by American and Iraqi troops led to the almost complete termination of the caliphate in 2008 (Hashim 2014).

In May 2010, Abu Bakr al-Baghdadi became the new leader of the Islamic State of Iraq. On July 22, 2012, he announced the return of the Islamic State in a public speech. The new stage of the Islamic State’s activity began on June 29, 2014, when the organization, after breaking the cooperation with Al-Qaeda, proclaimed itself a global caliphate. The activity of the global anti-terrorist coalition in Syria led to the displacement of ISIL and the transfer of its assets to Libya (Hashim 2014).

In the following years, ISIL was present in Libya and carried out propaganda activities and the acquisition of fighters. Although ISIL’s position has weakened after leaving Syria, the state of chaos in Libya has established a new foothold. By organizing three governors, taking into account the geographical division of Libya into Tripolitania, Cyrenaica and Fezzan, it expanded the caliphate outside Iraq and Syria. Although the coalition forces of Libya, supported by US aviation, weakened ISIL in 2016, they did not manage to completely eliminate the fighters who in central Libya began to rebuild their combat capabilities, constituting an important node in the global network of connections. The territory of Libya has become a support zone for organizations for terrorist attacks planned and carried out in Europe and Africa. In 2018, the organization was able to plan and conduct attacks in European cities, and training bases located in Libya provided training, tactical and logistical assistance to related groups and units in Tunisia, Algeria, Morocco and even Afghanistan. Libya has become a transit zone for terrorists traveling between the Middle East, North Africa and Europe, and where willing fighters are recruited from all over Africa. Grand Strategic Objectives for ISIL are still consistent:

- Expand the caliphate to encompass all Muslim lands.
- Provoke and win an apocalyptic war with the West.
- Assert unchallenged authority as a caliphate (Estelle 2017)

ISIL’s activities in Libya in the context of its strategic goals have a number of consequences that have a significant impact on the security of the EU and Europe as a whole. From the organization’s point of view, the most important consequence is the construction of the operational base and the return of the FTF, who joined ISIL in Syria and Iraq in the past, to their countries of origin. These threats may increase as ISIL strengthens and develops in Libya.

ISIL and Al-Qaida equally want to create a caliphate. Both organizations set themselves the goal of fighting the infidels, although ISIL extended the definition of the infidel to other branches of Islam besides Sunni. It also perfectly uses contemporary media to reach recipients. An example is the use of the Internet to attract not only native Muslims as their fighters. Successfully carried out propaganda allowed the acquisition of native citizens of Western countries to proclaim their ideology through the struggle and death of innocent people just because they are not Sunni and do not belong to the organization. Both groups are responsible for terrorist attacks in Europe, also ISIL has kidnapped representatives of Western countries, and presented spectacular and violent mass executions on the Internet.

The organizations would gladly eliminate each other to take over both material and personal resources, although in the case of Al-Qaeda the tendency to some form of cooperation for the benefit of unify the global Salafi-jihadi movement is more evident. The main purpose of ISIL is to reestablish the territorial caliphate in Libya, lost in Syria, mainly to preserve safe haven there and rebuild fighting force and command structure.

The fact that Libya remains a fragile country does not help to reduce, limit or eliminate activities of the organizations. Civil war, rivalry of the clans, lack of central government and strong state institutions such as army, police, judicial system brought the country on the verge of existence and is responsible for exporting migrants and FTFs to Western countries.

In May 2017, the US Director of National Intelligence Dan Coats concluded in congressional testimony that *“Europe will remain vulnerable to terrorist attacks, and elements of both ISIL and al Qaeda are likely to continue to direct and enable plots against targets in Europe”* (Coat 2017).

## Foreign terrorist fighters – Who are they

FTFs are not a new idea, but for the first time the jihad ideology has become attractive to people from Western civilization on such a large scale. The mere fact of the arrival of foreign fighters in a country where a civil war or a terrorist organization is active has often been used in the past. This took place, for example, in Afghanistan, where fighters from the Caucasus and the Arabian Peninsula came to fight the Soviet army in the 1980s. In the 1990s, when former Yugoslavia was falling apart, Caucasus fighters supported Muslims fighting throughout the Balkans. A similar situation took place in 2003 in Iraq, when fighters from Afghanistan and the Caucasus came to fight the US. It should be emphasized, however, that in those cases Muslims came to fight the infidels, supporting their Muslim brothers, so the situation was slightly different. ISIL managed to win over to its war citizens of Western countries who, fascinated by their ideology, were ready to convert to Islam and kill their kinsmen.

According to UN data, 40,000 fighters, foreigners from over 110 countries came to Syria and Iraq to fight for ISIL. With the displacement of ISIL from the territory occupied by the organization and the termination of the resulting caliphate, the US called for the admission of hundreds of men, women and children from the Middle East who were detained in the region due to ongoing fighting (Cook, Vale 2018). However, return to the country of origin is not so easy, because many countries are reluctant to re-admit these people to their territory.

The International Center for the Study of Radicalization and Political Violence at King’s College in London, based on official reports and research conducted by scientists published that by 2017 there were 41,490 fighters, including 32,809 men, 4761 women and 4640 children from 80 countries (Cook, Vale 2018). In the context of the main methodological assumptions and the purpose of the article, it is important and shocking that there were Europeans from Western Europe among the fighters arrived in the number of 5994. The most from the Baltic Sea countries came from Germany, about 1000. The second country whose citizens left for Middle East to fight was Sweden from which about 300 jihadists came. The ISIL ranks also include citizens of Denmark, Finland and Russia as well as Norway, although this country is not on the shores of the Baltic Sea, it is included in the countries of the Baltic region.

The loss of the caliphate had to change ISIL’s strategy and tactics. While in the period of growing popularity in 2014 to 2017, the leader Abu Bakr al-Baghdadi urged Muslims from all over the world to come to the caliphate, then after 2017 his rhetoric changed, encouraging him to stay in the countries where they live and organize terrorist attacks on the most sensitive targets in these places. Thus, terrorist activities have become the responsibility of every Muslim, and this reflects the changed, or rather adapted to the current realities, propaganda of the group, which no longer focuses on the caliphate, but increasingly calls for attacking Western targets (Neumann 2018).

The countries of the European Union and the whole of Europe must reckon with the fact that, in the light of this propaganda, they will become the object of intensified attempts and even terrorist attacks. Foreign fighters would play quite a substantial role in ISIL transformation. Especially, the FTFs with their intentions to pursue the legacy of the so called Caliphate. This could give a notorious boost to the homegrown extremism potential of the Euro-Mediterranean area (Kasapoglu 2018) including the BSR and maritime transport. The experience of recent years from actions that have taken place in France, Belgium, Great Britain or Germany allows stating that they do not have to be long and meticulously planned attacks, as Al-Qaida used to do. That means “an elevated terrorism threat posed by radicalized convicts, returned FTFs and other returnees who have direct ties to the legacy of the Islamic State. These may be opportunistic actions carried out by young militants from Muslim families, but the most dangerous may be FTF activities.

The threats associated with the operation of such fighters are multi-faceted. In the context of security in the Baltic Sea, the most likely scenario is the return of FTF to the countries of origin, where they will be able to travel freely with their passports around their country and the entire EU. Their activities can take the form of organizing a terrorist network in the country, planning a terrorist attack or using their experience in training young fighters (OSCE 2018). Regardless of which of the above-mentioned actions will be taken, they will be a real threat to the security of Western society. Statistics regarding the number of militants with EU passports who returned to their homelands are worrying, and are presented in Table 1.

**Table 1:** The number of FTF fighting in Iraq and Syria who returned to their countries of origin

Country	Total number of fighters	Number of fighters who returned	Status for month/year
EU	5000	1200	04/2016
Germany	915	300	03/2017
Sweden	300	106	09/2016
Denmark	145	67	02/2017
Norway	90	30	09/2016
Finland	80	43	02/2017
Russia	3417	400	03/2016

Source: Barrett 2017

The analysis of the data in the table shows that the countries in the BSR to which the largest number of militants returned are mainly Germany, Sweden and the Russian Federation. However, the author believes that fighters returning to Russia will focus on the Caucasus, as they are mainly Chechen fighters, not Russians. They will be a challenge for Russian security services, but their activity will not focus on the relatively small area of access to the Baltic Sea in the Gulf of Finland and the Kaliningrad Oblast. Other terrorists can get into EU countries with a mass of illegal immigrants who have been coming to the EU since the beginning of the Arab Spring in Libya.

The civil war in Libya, which began in 2011 and continues to date, has made its territory an oasis for terrorism and organized crime dealing in the smuggling of people, weapons and drugs. The funds obtained in this way support the activities of terrorist organizations. The security void created in Libya was quickly used by al-Qaeda and ISIL. Both organizations have powerful influence in Libya and although they do not permanently occupy areas, as was the case with ISIL in Iraq and Syria, they interact with local authorities and have the ability to further inspire terrorists with the help of Internet propaganda and to act with the help of FTFs returning to Europe.

## Ferry and cruise ships’ intensity in the Baltic Sea

BSR countries almost 100% belong to the EU, with the exception of the Russian Federation, which allows us to state that the Baltic Sea is almost an internal sea of the EU. Sea transport that takes place via sea routes is of great importance for European trade, because almost 90% of exports from the European Union and 40% of internal freight is carried out by sea (Urban 2018). The BSR plays a significant role for the Baltic States as well as for their external exchange. The Baltic Sea is one of the most heavily trafficked seas in the world, accounting for up to 15% of the world’s cargo transportation. It has also been known as difficult area for shipping for a long time. On one hand narrow straits, multiple islands and shallow waters do not leave a lot of space for navigation and on the other hand can create favorable conditions for organizing a terrorist attack which can possibly have a great impact on the BSR economy. As there are about 2000 ships in the Baltic marine area at any given time the possibility to choose the most suited vessel is very high. There are about 400 sea ports in the region of which 90 are of international importance. Between these ports commercial and passenger traffic is sailing throughout the year. For the purpose of this article, passenger traffic would be the most interesting.

Although according to International Maritime Organization (IMO) only 6% of ships in the Baltic Sea are passenger ships, they are important for ordinary or even daily transport, economy of the area in general and in terms of tourism business. For many Baltic ports and regions, cruise tourism is already or can become an important driver for increased attractiveness and an important source of income not only for the port, but also for local and regional communities. Most of the passenger traffic is the result of ferries but over the past three years, there has been a significant raise in cruise lines that the ports of the Baltic Sea are added to their routes, which increases overall passenger traffic (Kowalczyk 2018). Even though the cruise industry in the Baltic Sea is seasonal, due to climate and ice conditions, the activity lasts from early Spring till late Autumn with most calls in summer months. All of the BSR countries are tourist destinations and they constitute the largest cruise market in Northern Europe. Passengers of cruise lines are willing to explore UNESCO World Heritage Sites during short visits in Baltic ports. Although the number of vessel calls has remained rather stable over the last 15 years, the number of passengers has increased more than four times: from over 1 million passengers in 2000 to nearly 5,5 million in 2018 (Kłopott 2017, Meyer 2016). In fact it should be noticed that passenger transport related to ferry services has declined while the European cruise ship industry shows a strong upwards trend.

The ports have been hosting increasing number of larger vessels carrying 3 up to 4 thousand tourists ready to visit port cities and nearby vicinity every year (Urbanyi-Popiołek 2019). The region itself has been considered as historically attractive but also fairly safe and secure in terms of terrorist activities. It should be noticed that cruise shipping has become recently one of the major segments of contemporary tourism in general. The development of the cruise industry in the BSR is illustrated by the data of the top 10 ports in the region in 2019 presented in Table 2.

**Table 2:** The top 10 cruise ports of call in 2019

Country	Port of call	Number of ships visiting	Number of passenges
Denmark	Copenhagen	350	1,134,878
Estonia	Tallinn	338	656,000
Finland	Helsinki	303	605,000
Sweden	Stockholm	287	656,000
Russia	St Petersburg	262	645,000
Latvia	Riga	81	69,000
Poland	Gdańsk	60	22,411
Sweden	Gothenburg	59	110,000
Poland	Gdynia	54	118,000
Lithuania	Klaipeda	51	68,000

Source: Copenhagen Cruise Port 2019; The Maritime Executive 2019; Luty 2020

The statistics show that there were almost 4.3 million passengers traveling onboard of cruise ships and visiting the cities or close historic places and other attractions in 2019. Statistics on maritime passenger transport and tourism are not considered together and it should be noted that maritime tourism on cruise ships, taking as mentioned above, on average from 3 to 4 thousand passengers, brings certain revenues to the state economy, regularly visited by ships in the period from April to October. Revenues should be considered in a multifaceted way, starting with port fees for entering and servicing the vessel in the port, through a whole range of services related to serving tourists who spend a day or two in a given city, not mentioning the fact that satisfied tourists are the best ambassadors of the region.

The predictions published at the end of 2019, according to operators’ forecast, the growth trend would continue in the coming years, and the number of tourists visiting local destinations could reach nearly 7.6 million by 2025.

As at the end of 2017, the Baltic ferry fleet included 63 services on the Baltic Sea, operating 116 different ferries (Urban 2018). Cruise ferries, ships that combines the features of a cruise ship with a roll-on/roll-off ferry, they are also known as ro-pax for their combined freight vehicles and passenger design, cruise ro-ro ferries which are large conventional ferries named for the ease by which vehicles can board and leave and fast ro-pax ferries, conventional ferries with a large garage intake and a relatively large passenger capacity (Johnsen 2018). Ferry traffic goes through 71 ports within the borders of the BSR, with more than half of them in Sweden, Denmark and Finland.

Unfortunately, the volume of ferry traffic related to passenger and freight transport on the Baltic market is not possible to calculate. In previous years, most carriers published monthly data, hence it was possible to estimate preliminary results on most lines. Since 2016, some operators - for example TT-Line, Stena Line - stopped providing not only monthly data, but also did not publish annual results or published incomplete data (Urban 2018).

Tallink Silja is perhaps the largest ferry operator in the Baltic Sea, which operates between Estonia, Sweden, and Finland. They compete with Viking Line, one of the largest ferry operators with major routes within the Baltic Sea, with a focus on connecting Scandinavia. Finn Lines is probably the third large operator with routes, linking Germany to Finland and Sweden. They have a larger focus on cargo and those travelling with cars/trucks. Stena Line, very popular in Poland, offers many ferry routes in the lower part of the Baltic Sea. It offers a lot of ferries, especially between Germany/Poland and Sweden. DFDS is one of the largest passenger, cargo, and logistics companies in Europe. Their passenger routes on the Baltic Sea are a bit offbeat, but good for those with cars especially who are looking to make a crossing of the Baltic Sea, and to avoid traffic of the larger cities (Ferry Scan 2019).

Ferry transport is particularly important for the economy of BSR countries, which thus ensure a fast flow of goods transported, whether by trucks, tractor units or rail. The fact of relatively fast ferry transport, e.g. at night crossing, between the Baltic countries makes it a very attractive way of moving goods both in relation to the costs incurred in time and certainly competitive in relation to air transport. Its destabilization will cause measurable losses for the enterprise and the state, and thus indirectly for the whole region.

The presented data indicate that maritime transport in the BSR in relation to passenger and partially freight transport is a significant contribution to the development of the region and its economic security, which translates into a high standard of living in the BSR countries. Unfortunately the beginning of 2020 and the COVID-19 pandemic in Europe and the whole world has changed the situation drastically for tourism, cruise and ferry industry. The situation could potentially be worse for cruise ships as most of the internal and external borders within the EU and the world in general are being closed. Hopefully all the measures taken to master and eventually suppress the pandemic will work out and ferries lines will be operating again no later than at the beginning of summer season. Unfortunately the cruise industry season 2020 may be lost. At the same time administration and services responsible for security should be aware of possible activities of terrorist organizations, especially FTFs.

## The rising threat of a terrorist attack on passenger transport

The potential increase in terrorist activity in the BSR, primarily focused on ferry passenger transport or cruise ships visiting the region from outside the Baltic Sea, is in the author’s opinion associated with the return of FTF to the countries of the region from the war in Syria and Iraq. The above assumption was based on several premises that can be related to the analyzed area.

Presenting the individual bases of assumptions, the author first of all refers to general information about BSR, which has an impact on her assessment. Due to its geographical conditions, the Baltic Sea may become a theatre of terrorist activities because it is a closed or semi-closed body, which means difficulties in exchanging waters with the Atlantic Ocean, which is the northern arm. Such conditions have an impact on the effectiveness of a potentially effective terrorist attack, which, depending on its location, course and materials used, may cause a palpable ecological disaster in all countries of the region. These conditions have one more element of danger, the Baltic Sea has been known as difficult area for shipping for a long time as narrow straits, multiple islands and shallow waters do not leave a lot of space for navigation. Dependent on season shipping also has to deal with rough weather conditions such as intensive storms during autumn, strong currents in the straits and icy waters during winter. In addition to these natural restrictions to shipping, new interests have arisen in the past decades. Most relevantly is the vast need for space for offshore wind farms (Meyer 2016). In the event of a terrorist attack, communication lines on the Baltic Sea may be blocked and units, due to their immersion and transported cargo, will not be able to change route. The presence of numerous islands, bays and straits, especially on the north and west coasts, can favor terrorist activities. It cannot be ignored at this point that the Baltic Sea is also a well-monitored reservoir, nevertheless computers responsible for Baltic traffic activity are served by people who may be recruited to a terrorist organization or simply bribed. The human aspect discussed here is also important for another reason.

The Baltic Sea countries are inhabited by a large Muslim Diaspora, which could potentially become a base for future terrorist activities. Table 3 presents the number of Muslims in some countries of the region, but attention is drawn to countries such as Germany, Sweden, Denmark and obviously Russia because of a significant size of the Diasporas. The author does not suggest that every Muslim is involved in terrorist activities, but certainly the fact of the numerous Diasporas may favor such activity by raising the level of potential threat.

**Table 3:** Population of Muslims in the BSR in 2016

Country	Total population	Muslim population	%
Denmark	5,800,000	240,000	4,1
Germany	82,800,000	4,802,000	5,8
Russia	147,300,000	27,990,000	19
Sweden	10,200,000	499,800	4,9
Finland	5,500,000	148,500	2,7
Poland	38,000,000	25,000–40,000	0,1

Source: Pew Research Center 2017

Disturbing is the fact that it is from three countries: Germany, Sweden, Denmark that the most fighters went to fight for ISIL in Iraq and Syria, and also most of them returned home after the breakup of ISIL and partial transfer of assets to Libya. These are citizens with European passports who can move freely not only in their country but throughout the EU and as presented in Part 2 may pose a serious threat.

Combining FTF activity with ferry transport in BSR, it should be noted that it can be used either to move freely between countries or become the target of the attack itself. Numerous ferry connections from companies such as DFDS Stenaline, Finnlines and Tallink Silia Line can become attractive to terrorists. First of all, e.g. DFDS offers connections between local and not-so-attractive tourist destinations, especially for truck transport, which creates favorable conditions for the logistic base of terrorists who will avoid popular and security-controlled ports. In contrast, ports attractive for tourists with high passenger traffic may become the target of the attack itself. It can be carried out by means of a truck, a passenger car unloaded with explosives, which will enter the ferry, and then, after leaving the port, in the sea, in a properly selected place will be detonated causing a catastrophe and sinking of the ferry. Despite the threat of terrorist activities, ro-ro and ro-pax ferries leave terrorists the opportunity to organize an attack. From information obtained during talks with a security expert, security director at the Port of Gdynia, cpt. N. PhD. Józef Zawadzki, in the first five months of 2019 there were several incidents, fortunately false, in which the anonymous caller reported by phone about explosives placed on the ferry. Each time an action is carried out to thoroughly check the ferry and vehicles on the ferry, which causes measurable financial losses both for the port that carries out such operations and for the carrier who is delayed and for the passengers themselves.

BSR terrorist attacks can be targeted at the cruise ship region in large numbers. The presence of tourists of different nationalities on such a unit can be attractive to jihad fighters who can attack both while crossing the sea and in the port itself. The favorable factor in both cases is the planning of the crossing or voyage, which allows you to plan the attack and use all the advantages and eliminate potential disadvantages.

Another important issue, which should be taken under consideration, is the fact that FTFs may want to use all kinds of civilian drones to help them to organize or to perform an attack. Drones are relatively small, civilian unmanned, flying vehicles, able to carry up to 5–6 pounds of load on average altitude up to 300 m. They can be operated by a trained person by radio waves. The major issue in terms of such drones is the fact that the regulations concerning the usage of them in different public spaces, are not consistent and in some countries are still under some works (for example in Poland). There is also the lack of understanding of importance such regulations by many people. It cannot be ignored that the accessibility and relatively low prices of this technology makes them very popular.

The drones can be used in different ways either for smuggling not too heavy loads, by dropping a package with weapons on board a ferry or cruise ship. They could also be used to attack a vessel. This could be done by placing some explosives in a package. This might cause death of some passengers but also panic on board. In edition it may be designed to immobilize a vessel on an approaching track to a port, in straits, channels or canals. Long and short term consequences could be destructive for regional economy.

Each attack carried out on passenger units is primarily related to human casualties. The Baltic Sea, especially during winter storms, is a difficult sea to carry out rescue operations, as demonstrated by the previous sea disasters that took place in the Baltic, e.g. the sinking of the Polish ferry *MF Jan Heweliusz* in January 1993, in which 55 people were killed. That is why the time, place and object of the attack, carefully chosen by terrorists, may intensify its tragic effects. It cannot be ignored that at present ro-ro and ro-pax ferries are part of uninterrupted trade and economic exchange of BSR countries, and the attack on this transport will also translate into economic and political effects. The neighborly relations of the BSR countries may be tarnished by mutual accusations of not providing an adequate level of security.

It is a mistake to say that terrorist organizations such as ISIL or Al-Qaeda have been broken up and lack the ability to organize attacks. Propaganda, which is preached all the time on the Internet in the case of ISIL, inspires and even calls on jihadists to organize various attacks. *Inspire* Magazine still remains Al-Qaeda’s most efficient tool to motivate to organize terrorist attacks. It cannot be forgotten that supporters and fighters in many countries actively remain a global threat through its dormant “cells” present around the world. Considering the above, the author is convinced that the level of threat to ferry transport in the BSR is real due to the returning FTF, although not as high as in the first decade of the twenty-first century. Their frustration, dissatisfaction with the situation they found themselves in may sooner or later find a discount in the organization of a terrorist attack.

## Conclusions

On September 11, the world celebrated the 19th anniversary of its tragic terrorist attacks. In the same year, in a report published in January 2019 by the UN regarding The Analytical Support and Sanctions Monitoring Team submitted pursuant to resolution 2368 (2017) concerning ISIL, Al-Qaeda and associated individuals and entities, we read that: “*ISIL continues to pose a significant threat in Europe despite its diminished ability to direct attacks. There have been fewer ‘lone actor’ attacks recently, which suggests that the ability even to inspire such attacks may be declining. Nevertheless, there has been a recent re-emergence of communication between ISIL command and control, and individuals in different European countries. FTF remain an issue, illustrated by the major terrorist attack plan foiled in the Netherlands in September 2018.*” The EU must be prepared to the possibility of new terrorist attacks on EU soil, including the BSR, and growing concerns over foreign fighter returnees, that pose threats to civilians, our economies. The fact that the jihadists form ISIL remain stateless and eradicated from Iraq and Syria does not mean that their adherents have been killed nor their ideology debunked.

Having this knowledge some steps should be taken to prevent preparations or performance any successful attacks. The measures should be considered in two groups: technical and organizational. The technical ones can be connected with preventive measures against COVID-19.

Technical measures:

- Every border crossing in BSR ports should be equipped with high quality cameras able to recognize faces of passengers (e.g. people wanted for terrorism and criminal activities). They could be combined with an infrared component used for fever detection against COVID-19.
- It would be crucial to bring up to date all databases regularly, particularly with records of people wanted or connected with terrorist activities, especially TFTs. Such databases should be available to all national security services (e.g. the police, border guards). This could help to identify them quickly and stop not only terrorists, but also criminals.
- All of the border crossings in BSR ports should have necessary equipment for detection of forged documents, which obviously help TFTs to move freely from country to country even if they have records in databases.
- In terms of drone threats all BSR ports should be classified as restricted areas and equipped with counter-drone systems. There are several systems available on the market but the type of detectors used, their technical parameters, the impact of the environment in which the device would work (atmospheric factors, time of day e.g. night), interaction with nearby devices (e.g. audio interference) determine the information we expect from a detector, i.e.: the detection distance of the intruder, the occurrence and the number of false alarms, and often at all the possibility of its operation (e.g. audio, video detection, thermal vision, radar or radio waves). This should be the subject of individual expectations and possibilities but most of all it depends of awareness of the management board of every port.

Organizational measures:

- Permanent vigilance on the border crossings is one of the most important elements of the security in general and in such environment as maritime infrastructure (a post, a vessel) especially.
- The personnel of security services should be well trained. Their qualifications and skills should be constantly improved during national and international exercises and training.
- The EU and every single country should intensify all the works connected to drone legal regulations. Clear law can be helpful in banning drones in port’s space and also in punishing any acts of violence in this matter.

If we take our security seriously we cannot ignore any possibilities that can work in favor for terrorists. According to the author, both Al-Qaeda and ISIL are still capable of terrorist activities, and the fact that the presence of already trained and seasoned FTFs with European passports in the BSR countries creates even better conditions for active actions and increases their likelihood. The Baltic Sea and the activity of its inhabitants have not been the target of terrorists so far, but there is always the first time. 19 years ago, no one expected a passenger to be hijacked and directed to the World Trade Center, and terrorists chose this scenario. Attacking passenger transport in the Baltic Sea may prove to be an attractive and spectacular target that can have unimaginable and multi-faceted consequences due to potential victims first of all and the slowdown or even local economic collapse. 2020 has proved to be a very tragic and demanding year all over the world, unfortunately terrorists may want to use our weakness in their favor. EU and BSR governments should be prepared for the possible occurrence of such an attack because, unfortunately, terrorists decide which target they choose, when they attack and how.
